# Bacterial and fungal communities within and among geographic samples of the hemp pest *Psylliodes attenuata* from China

**DOI:** 10.3389/fmicb.2022.964735

**Published:** 2022-09-06

**Authors:** Litao Guo, Chao Tang, Chunsheng Gao, Zhimin Li, Yi Cheng, Jia Chen, Tuhong Wang, Jianping Xu

**Affiliations:** ^1^Institute of Bast Fiber Crops, Chinese Academy of Agricultural Sciences, Changsha, China; ^2^Department of Biology, McMaster University, Hamilton, ON, Canada

**Keywords:** *Psylliodes attenuata*, microbial community diversity, 16S rRNA, ITS, hemp

## Abstract

The hemp flea beetle *Psylliodes attenuata* (Coleoptera: Chrysomelidae: Psylliodes) is a common pest of *Cannabis sativa*, including cultivars of both medicinal marijuana and industrial hemp. Both the larval and adult stages of this beetle can cause significant damages to *C. sativa*, resulting in substantial crop losses. At present, little is known about the bacterial and fungal community diversity among populations of this pest insect. In the present study, we obtained *P. attenuata* samples from nine field sites representing broad industrial hemp productions in China and analyzed their microbial communities using DNA metabarcoding. Bacterial sequences of all the samples were assigned to 3728 OTUs, which belonged to 45 phyla, 1058 genera and 1960 known species. The most common genera were *Rickettsia*, *Wolbachia*, and *Candidatus_Brownia*. Fungal sequences of all the samples were assigned to 910 OTUs, which belonged to 9 phyla, 308 genera and 464 known species. The most common fungal genera were *Cladosporium*, *Cutaneotrichosporon*, and *Aspergillus*. Principal coordinate analysis revealed a significant difference in the bacterial and fungal community structure among the nine *P. attenuata* populations. Understanding the microbial symbionts may provide clues to help develop potential biocontrol techniques against this pest.

## Introduction

Being one of the world’s oldest crops with significant economic and medicinal importance, *Cannabis sativa* L. (cannabis) has attracted an enormous amount of attention and become one of the most commonly cultivated plants worldwide (Deguchi et al., 2020; Gao et al., 2020). Based on the statistics from Organization for Economic Co-operation and Development (OECD), industrial hemp is grown in more than 130 countries with 94 certified hemp varieties for large-scale productions of food, textiles and building materials. Similarly, *C. sativa* is of significant medical and recreational interests due to its productions of the psychoactive compound D-9-tetrahydrocannabinol (THC) and/or other cannabinoids such as cannabidiol (CBD). CBD and THC have shown pharmacological properties in the treatment of mood disorders, pain, cancer, diabetes, and inflammatory and neurodegenerative diseases (Andre et al., 2016; Irakli et al., 2019).

The hemp flea beetle, *Psylliodes attenuata* (Koch) 1803 (Coleoptera: Chrysomelidae: Psylliodes), is among the most common pests that can cause severe crop losses in *Cannabis* production areas (Goncalves et al., 2019). *P. attenuata* is geographically broadly distributed across Eurasia, from the United Kingdom to France, the Middle East, eastern Siberia, Japan, and China (Goncalves et al., 2019). The larvae and adult of the hemp flea beetle can damage hemp crops, with adults causing more damages than grubs. The larvae feed on hemp roots, and the adult feed on apical leaves, flowers, and seeds of hemp (McParland et al., 2000). Many flea beetles are polyphagous, but the hemp flea beetle is mostly an oligophagous insect, feeding on hemps (Zhao et al., 2018; Deguchi et al., 2020). Indeed, hemp flea beetle has been suggested as a candidate biocontrol agent for eradicating illegal marijuana growth due to its obligatory feeding on hemp (Hall, 1999).

Insect microbiota have attracted a lot of attention because they have shown to provide a diverse range of effects on insects, from nutrition, physiology, to behavior. For example, insect microbiota are known to detoxify noxious secondary metabolites and xenobiotics, such as terpenes (Berasategui et al., 2017), caffeine (Ceja-Navarro et al., 2015; Summers et al., 2015), nicotine (Brandsch, 2006), and insecticides; defending against parasites and other pathogens; generating and relaying signals among hosts; improving behavior and immunity; and producing nutrients to supplement host’s diet and to enhance their digestion (Dillon and Dillon, 2004; Ezemwa et al., 2012; Wu et al., 2016). In recent years, a growing number of studies have cataloged and characterized microbial communities, particularly in bees (Moran et al., 2012), fruit flies (Erkosar et al., 2017; Bing et al., 2018), termites (Rossmassler et al., 2015), silkworm (Chen et al., 2018), whiteflies (Santos-Garcia et al., 2020), and beetles (Kudo et al., 2018). For example, Zhang et al. (2022) found that a normal gut microbiota is required for olfactory learning and memory abilities, mainly by regulating tryptophan metabolism, with host-specific *Lactobacillus* strains enhancing memory by transforming tryptophan to indole derivatives that activate the host aryl hydrocarbon receptor in honeybee (Zhang et al., 2022).

In a previous study, we analyzed the genetic diversity and geographic structure of *P. attenuata* in hemp fields in China based on mitochondrial COI gene sequences. Our analyses revealed that geographic separation among the regional populations accounted for 58% of the total genetic variance, while within-local populations accounted for 42% of the total observed genetic variance (Guo et al., 2020). However, the significance of the observed genetic diversity and geographic differences remains unknown. In this study, we analyzed the bacterial and fungal communities associated *P. attenuata* to investigate whether the different geographic populations of *P. attenuata* contain distinct bacterial and fungal communities. In addition, we were interested in whether there were common shared bacterial and fungal species among the diverse *P. attenuata* populations. Commonly shared microbes among geographic diverse hosts would suggest their potential key functions to these pests. In contrast, geography-specific patterns would suggest unique microbial communities for individual geographic regions. Such discoveries could potentially help us design novel strategies to control and prevent the damaging effects of these pests on hemp crops.

To achieve our objectives, we broadly sampled hemp flea beetles in four hemp-growing regions in China where nine *P. attenuata* populations were collected. We then obtained data on microbial communities inside these flea beetles using high-throughput next-generation DNA sequencing. Finally, we analyzed the alpha diversity, the beta diversity, and predicted functions of the microbial community. The observed data were discussed in the context of general microbial diversity in insects, their potential roles in the biology of the hemp flea beetles, and implications for developing future control and management strategies against these pests.

## Materials and methods

### Sample collection and identification of *Psylliodes attenuata*

**FIGURE 1:**
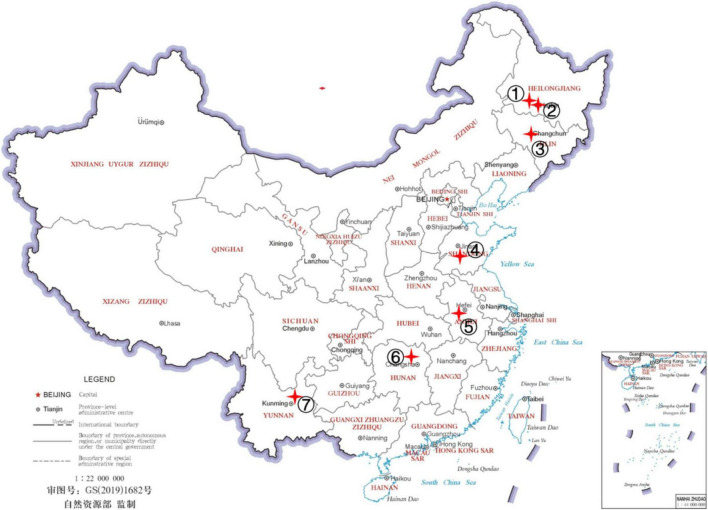
Collection localities for *Psylliodes attenuata*. The map was downloaded from http://bzdt.ch.mnr.gov.cn. The locations of sampling sites are noted with numbers and red cross on the map. The details of *P. attenuata* collections were shown in Table 1.

For this study, we broadly sampled hemp flea beetles in four hemp-growing regions in China: northeastern China (including Heilongjiang and Jilin provinces), central-east China (Shandong and Anhui provinces), south-central China (Hunan province), and southwestern China (Yunnan province). Nine sites within the four regions were sampled and the site information is presented in Figure 1 and Table 1 (Guo et al., 2020). The collected adult beetles were soaked in 99.7% anhydrous alcohol, and then surface-cleaned with PBS buffer for three times, placed in a sterile centrifuge tube, frozen with liquid nitrogen for more than 1h and stored at −80°C. These beetles were identified as hemp flea beetles based on their morphological features and mitochondrial COI sequences (McParland et al., 2000; Guo et al., 2020). At each of the nine sites, we obtained 20 flea beetles for analyses, with each flea beetle collected from a different hemp plant. The 20 flea beetles from each site were randomly grouped into four samples as biological repeats, with each sample containing five beetles to ensure that sufficient microbial DNA could be extracted for analyses from each sample. A total of 36 samples were collected and analyzed in this study.

**TABLE 1:** The details of *Psylliodes attenuata* collections.

Number	Province	Site	Latitude/longitude	Host cultivar	Date collected	Population code
**➀**	Heilongjiang	Daqing	46.67/125.23	Qingma Nos. 1 and 2	September 2019	DQ
**➁**	Heilongjiang	Harbin	45.59/126.44	Long Cannabis No. 3	September 2019	HRB
**➂**	Jilin	Changchun	43.72/125.09	Fenma No. 3	September 2019	JL
**➃**	Shandong	Tai’an	35.97/116.97	Zhong Cannabis No. 5	August 2019	SD
**➄**	Anhui	Wangqiao Village	31.67/116.357	Wan Cannabis No. 1	July 2019	AH1
	Anhui	Lu’an	31.806/116.519	Wan Cannabis No. 1	July 2019	AH2
	Anhui	Zhangwan Village	31.70/116.358	Wan Cannabis No. 1	July 2019	AH3
**➅**	Hunan	Shijihu	28.8/112.36	Zhong Cannabis No. 1	August 2019	HN
**➆**	Yunnan	Qujing	25.85/103.75	Yunma No. 7	September 2019	YN

### DNA extraction and PCR Amplification

Microbial community genomic DNA was extracted from each of four samples at each of the nine sites using the E.Z.N.A.^®^ soil DNA Kit (Omega Bio-Tek, Norcross, GA, United States) according to manufacturer’s instructions. The DNA extract was checked on 1% agarose gel, and DNA concentration and purity were determined with NanoDrop 2000 UV-vis spectrophotometer (Thermo Fisher Scientific, Wilmington, DE, United States). The 16S ribosomal RNA (rRNA) gene spanning hypervariable regions (V3−V4) and fungal internal transcribed spacer 1 (ITS1) region were respectively amplified using the following primer pairs: 338F/806R (5′-ACTCCTACGGGAGGCAGCAG-3′ and 5′-GGACTACHVGGGTWTCTAAT-3′) and ITS1F/ITS2R (5′-CTTGGTCATTTAGAGGAAGTAA-3′ and 5′-GCTGCGTTCTTCATCGATGC-3′) by an ABI GeneAmp 9700 PCR thermocycler (ABI, CA, United States). The PCR amplification of 16S rRNA/ITS1 DNA fragments was performed as follows: initial denaturation at 95°C for 3 min, followed by 28/35 cycles of denaturing at 95°C for 30 s, annealing at 55°C for 30 s and extension at 72°C for 45 s, and single extension at 72°C for 10 min, and end at 10°C. Each PCR mixture contained 5 × *TransStart* FastPfu buffer 4 μL, 2.5 mM dNTPs 2 μL, forward primer (5 μM) 0.8 μL, reverse primer (5 μM) 0.8 μL, *TransStart* FastPfu DNA Polymerase 0.4 μL, BSA 0.2 μL, template DNA 10 ng, and finally ddH_2_O up to 20 μL. PCR reactions were performed in triplicate. The PCR products were extracted from 2% agarose gel and purified using the AxyPrep DNA Gel Extraction Kit (Axygen Biosciences, Union City, CA, United States) according to manufacturer′s instructions and quantified using Quantus™ Fluorometer (Promega, United States).

### Illumina MiSeq sequencing

For both the amplified 16S rRNA and ITS1 sequences, the purified amplicons from each of the 36 samples were separately barcoded and the 36 barcoded samples were pooled in equimolar and paired-end sequenced on an Illumina MiSeq PE300 platform/NovaSeq PE250 platform (Illumina, San Diego, CA, United States) according to the standard protocols by Majorbio Bio-Pharm Technology Co., Ltd. (Shanghai, China). The raw reads were deposited into the NCBI Sequence Read Archive (SRA) database (BioProject Number: PRJNA843905; sequence accession numbers SRR19449852-SRR19449887).

### Processing of sequencing data and statistical analysis

The raw 16S rRNA and ITS1 sequencing reads were demultiplexed, quality-filtered by fastp version 0.20.0 (Chen et al., 2015) and merged by FLASH version 1.2.7 (Magoč and Salzberg, 2011) with the following criteria. First, the 300 bp reads were truncated at any site receiving an average quality score of <20 over a 50 bp sliding window, and the truncated reads shorter than 50 bp were discarded, reads containing ambiguous nucleotide bases were also discarded; (ii) only overlapping sequences longer than 10 bp were assembled according to their overlapped sequence. The maximum mismatch ratio of overlap region was set at 0.2. Reads that could not be assembled were discarded; (iii) Samples were distinguished and sorted according to their barcode and primers.

The data were analyzed on the free online platform of Majorbio Cloud Platform^1^. Operational taxonomic units (OTUs) with 97% similarity cutoff (Stackebrandt and Goebel, 1994; Edgar, 2013; Xu, 2016, 2020) were identified and clustered using UPARSE version 7.1 (Edgar, 2013), and chimeric sequences were identified and removed. The taxonomy of each OTU representative sequence was analyzed by RDP Classifier version 2.11 (Wang et al., 2007) against the 16S rRNA database (Silva v138) and ITS database (Unite v8.0) (Kõljalg et al., 2013) using confidence threshold of 0.7. The curated OTU data in QIIME were used to calculate the alpha diversity indices by Mothur version 1.30.2, including Sobs, Chao, Ace estimators, Shannon and Simpson indices, respectively. Values were compared using one-way ANOVA Tukey test among samples, *P* < 0.05 was considered statistically significant. These analyses were performed with the Statistical Package for the Social Sciences, version 20.0 (SPSS, Chicago, United States). Bray–Curtis distance matrices were constructed using rarefied OTU abundance table and visualized in principal coordinate analysis (PCoA). Taxonomic heat maps were clustered by the average linkage method with the Bray–Curtis distance and generated by the R package. Both the total OTU count data and rarefied data were used for comparisons among samples. The functional of the bacterial and fungal community was respectively predicted by PICRUSt2 and FUNGuild.

## Results

### Comparison of bacterial communities across all samples

The composition of the bacterial communities within and among the nine geographic populations of the hemp flea beetle *P. attenuata* in China was investigated by high-throughput metabarcode DNA sequencing. Our sequencing generated a total of 1,887,854 reads of bacterial 16S rRNA genes with an average length of 412 bp. In total, 3728 bacterial OTUs were identified among the nine geographic populations. These bacterial OTUs belonged to 45 phyla, 1058 genera, and 1960 species (Table 2 and Supplementary Table 1). After rarefaction according to the lowest sample sequence number, 2933 OTUs were clustered, attributed to 42 phyla and 970 genera and 1751 species for the nine *P. attenuata* populations (Table 2 and Supplementary Table 2). Rarefaction curves from both the original sequencing data and rarefied data sets showed that the curves of all 36 samples approached asymptote, indicating that the amount of sequencing data was sufficiently representative of the microbial diversity within each of the 36 samples (Supplementary Figure 1).

**TABLE 2:** Summary of the 16S ribosomal RNA (rRNA) and internal transcribed spacer (ITS) read counts of bacteria across nine geographic populations of the hemp flea beetle *P. attenuata* in China.

Sample name	16S							ITS						
	Phylum	Class	Order	Family	Genus	Species	OTU number	Phylum	Class	Order	Family	Genus	Species	OTU number
AH1	27	58	147	235	403	572	724	3	18	39	78	111	148	208
AH2	23	43	91	183	322	417	478	5	21	44	84	115	156	219
AH3	20	39	100	146	234	295	339	3	15	31	62	74	92	128
DQ	27	64	152	250	446	631	750	5	18	33	57	80	99	159
HRB	23	38	101	157	265	351	388	5	17	32	51	62	78	114
HN	37	85	213	374	745	1274	2029	4	19	37	78	106	136	191
JL	22	37	83	136	205	260	286	3	19	42	68	86	118	167
SD	24	41	100	170	314	453	609	4	14	26	43	56	80	110
YN	16	29	74	116	179	222	243	4	16	32	56	77	100	136

**FIGURE 2:**
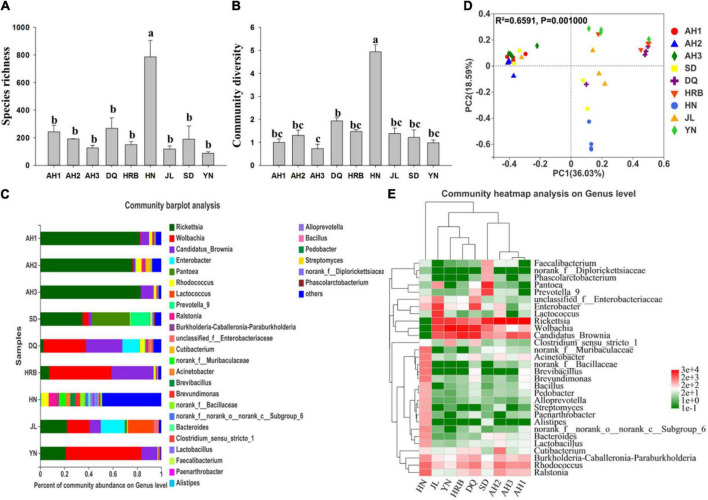
Bacterial community composition within and among nine geographic populations of the hemp flea beetle *P. attenuata* in China. **(A)** Histogram of species richness (number of OTUs). **(B)** Histogram of community diversity measured by Shannon index. Letters above each population indicate significant differences (one-way ANOVA, Tukey test, *P* < 0.05, see Table 3) in the mean values. **(C)** Relative abundance of bacterial genera in different populations. **(D)** Principal components analysis (PCoA) plots of bacterial communities based on the Bray-Curtis distance. Each symbol or color represents a sample. The significant differences in beta diversities were analyzed using Adonis analysis with 999 permutations, *P* < 0.05. **(E)** Heatmap showing the relative abundance of top thirty bacterial genera. Hierarchical cluster analysis was based on the Bray-Curtis distance with average method.

There were significant differences in bacterial species richness (observed OTUs) and community diversity (Shannon) with Alpha-diversity analyses between the HN and the other eight geographic populations (including AH1, AH2, AH3, DQ, HRB, JL, SD, and YN) (Figures 2A,B and Tables 2, 3). In addition, species richness of the HN population was the highest among the nine geographic populations (Figure 2A). At the phylum level, Proteobacteria and Bacteroidetes dominated the bacterial community across all samples but HN (Supplementary Figure 2). At the genus level, OTUs of the AH1 population were mainly annotated to *Rickettsia* (82.2%) and *Candidatus_Brownia* (7.04%); OTUs of AH2 were mainly annotated to *Rickettsia* (76.42%), *Cutibacterium* (4.67%), and *Rhodococcus* (4.5%); OTUs of AH3 were mainly annotated to *Rickettsia* (83.86%) and *Candidatus_Brownia* (9.81%); OTUs of SD were mainly annotated to *Rickettsia* (36.2%), *Pantoea* (33.21%), and *Prevotella_9* (13.11%); OTUs of DQ were mainly annotated to *Wolbachia* (35.73%), *Candidatus_Brownia* (30.48%), and *Enterobacter* (13.45%); OTUs of HRB were mainly annotated to *Wolbachia* (53.12%), *Candidatus_Brownia* (33.76%), and *Rickettsia* (6.69%); OTUs of JL were mainly annotated to *Rickettsia* (21.55%), *Enterobacter* (20.69%), *Lactococcus* (20.64%), *Wolbachia* (18.56%), and *Candidatus_Brownia* (9.34%); OTUs of YN were mainly annotated to *Wolbachia* (62.55%), *Rickettsia* (21.41%), and *Candidatus_Brownia* (12.81%) (Figure 2C). The similarities and differences in composition and structure of the bacterial communities among all geographic samples were revealed by Principal components analysis (PCoA). PCoA separated most field populations (ADONIS test with 999 permutations, *P* < 0.05) except the SD, JL, and DQ samples (Figure 2D).

**TABLE 3:** The diversity indices of bacteria and fungi based on 16S rRNA and ITS sequences respectively across nine geographic populations of the hemp flea beetle *P. attenuata* in China.

Types	Diversity indices	AH1	AH2	AH3	DQ	HRB	HN	JL	SD	YN
**16S**	**Sobs**	243.25 ± 48.87	190.75 ± 2.50	127 ± 16.97	268.75 ± 75.31	149.75 ± 20.82	787 ± 118.83	117.25 ± 24.52	189.75 ± 96.12	88.00 ± 9.35
	**Shannon**	0.99 ± 0.15	1.30 ± 0.23	0.73 ± 0.19	1.93 ± 0.14	1.47 ± 0.08	4.95 ± 0.29	1.38 ± 0.24	1.22 ± 0.32	0.98 ± 0.12
	**Simpson**	0.99 ± 0.15	1.30 ± 0.23	0.73 ± 0.19	1.93 ± 0.14	1.47 ± 0.08	4.95 ± 0.29	1.38 ± 0.24	1.22 ± 0.32	0.98 ± 0.12
	**ACE**	287.05 ± 69.30	205.26 ± 4.11	140.71 ± 17.64	307.77 ± 106.37	170.15 ± 21.76	813.65 ± 119.90	166.29 ± 18.47	277.79 ± 120.51	126.59 ± 7.57
	**Chao**	289.91 ± 72.72	204.10 ± 4.21	139.98 ± 17.87	306.85 ± 104.12	177.72 ± 25.75	815.82 ± 118.43	146.86 ± 22.05	263.32 ± 110.57	118.83 ± 8.39
	**Coverage**	0.9985 ± 0.0006	0.9993 ± 0.0001	0.9994 ± 0.0000	0.9986 ± 0.0009	0.9992 ± 0.0001	0.9983 ± 0.0001	0.9991 ± 0.0001	0.9981 ± 0.0008	0.9991 ± 0.0000
**ITS**	**Sobs**	87.50 ± 2.96	75.50 ± 15.57	46.00 ± 3.42	54.00 ± 8.77	44.75 ± 3.25	71.50 ± 7.38	72.00 ± 3.49	42.75 ± 13.11	51.50 ± 3.23
	**Shannon**	1.68 ± 0.18	2.61 ± 0.22	2.01 ± 0.46	2.68 ± 0.35	2.47 ± 0.23	2.50 ± 0.19	2.31 ± 0.06	1.55 ± 0.58	1.92 ± 0.53
	**Simpson**	0.43 ± 0.07	0.16 ± 0.04	0.33 ± 0.15	0.16 ± 0.04	0.16 ± 0.04	0.17 ± 0.03	0.23 ± 0.01	0.44 ± 0.20	0.36 ± 0.18
	**ACE**	99.46 ± 6.77	84.35 ± 12.55	52.95 ± 5.75	55.87 ± 8.54	46.11 ± 3.72	73.73 ± 7.54	75.16 ± 3.95	45.41 ± 13.81	52.42 ± 2.69
	**Chao**	99.74 ± 6.38	80.18 ± 16.01	52.56 ± 8.07	56.33 ± 8.74	46.25 ± 3.94	72.88 ± 7.65	75.50 ± 4.54	45.02 ± 14.39	52.40 ± 2.45
	**Coverage**	0.9997 ± 0.00005	0.9999 ± 0.00003	0.9999 ± 0.00004	0.9999 ± 0.00002	1.0000 ± 0.0003	0.9999 ± 0.00003	0.9999 ± 0.00002	0.9999 ± 0.00002	1.0000 ± 0.0003

We selected the bacterial genera with the top thirty abundance ratios and drew heat maps based on their relative abundances in different geographic populations (Figure 2E). From the overall distribution of the microbial community at different populations, *Rickettsia*, *Wolbachia*, and *Candidatus_Brownia* were the dominant genera (Figure 2E and Supplementary Figure 3) across most of the samples except the HN samples. *Pantoea* (33.21%) was abundant in SD, but the numbers were relatively small in the other populations. The abundance ratio of *Lactococcus* in JL was significantly higher than those in the other populations (Figure 2E). Overall, the bacterial communities from the three AH populations (AH1, AH2, and AH3 samples) were clustered together, with the SD sample being most similar to them among the non-AH samples. HRB, DQ, YN and JL populations clustered into a different group, while the HN populations were distinctly different. There was no genus in the HN population exceeding 6.30%.

### Abundance of bacteria across Samples

**FIGURE 3:**
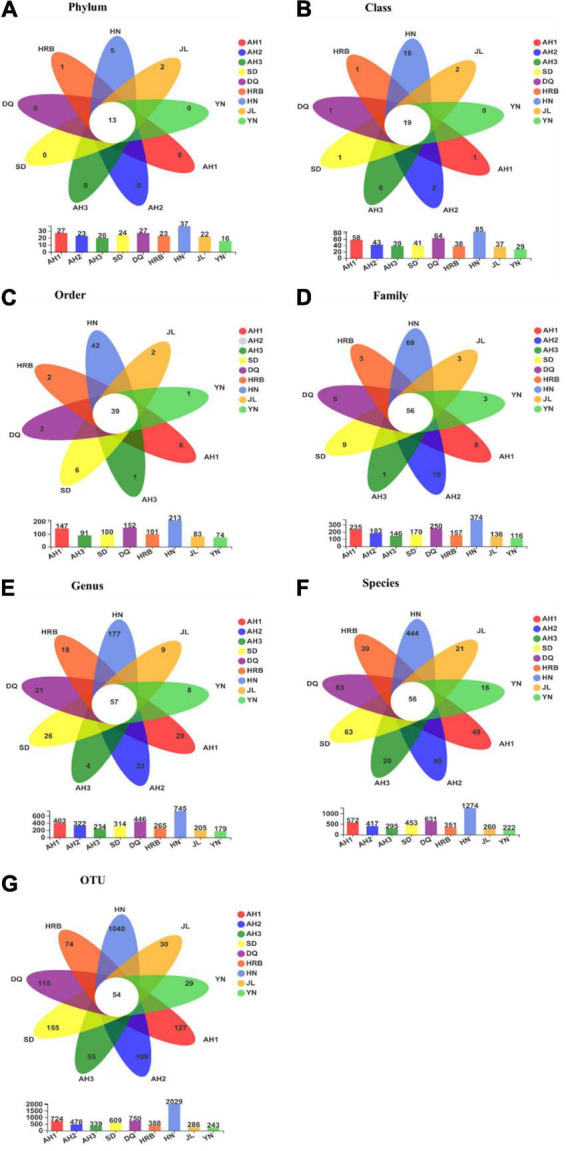
**(A–G)** Overlap among bacterial communities at different taxonomic levels among the nine geographic populations of the hemp flea beetle *P. attenuata* in China, with the number of species unique to each population in the petal and the number of species common to all populations in the center. Below is a bar chart of the total number of species in each population at selected taxonomic level.

Figure 2 shows the bacterial community overlap among the nine geographic regions of the hemp flea beetle *P. attenuata* in China at different taxonomic levels. At all taxonomic levels, the HN population had the highest richness (Figure 3). At the OTU level, there were 54 shared OTUs among the nine geographic regions, and the unique OTUs of AH1, AH2, AH3, SD, DQ, HRB, HN, JL, and YN were respectively 127, 109, 55, 155, 115, 74, 1040, 30, and 29 (Figure 3). At other taxonomic levels, there were 13 shared Phyla, 19 shared Classes, 39 shared Orders, 56 shared Families, 57 shared Genera, 56 shared known species, and 54 shared OTUs among the nine geographic regions (Figure 3).

### Bacterial community dissimilarity across samples

**FIGURE 4:**
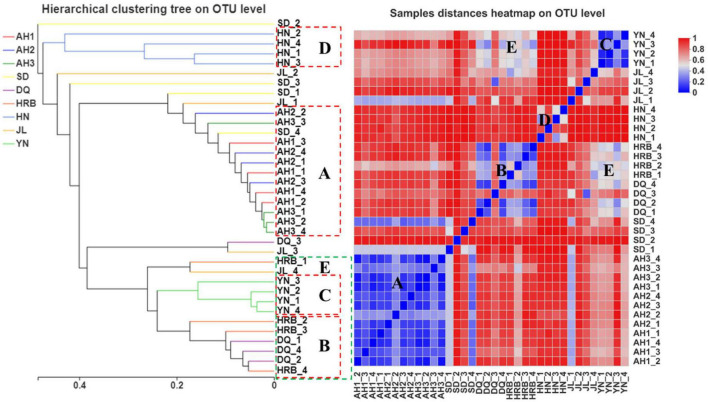
Bacterial community dissimilarity among samples from nine geographic populations of the hemp flea beetle *P. attenuata* in China. The picture on the left: analysis of unweighted pair-group method with arithmetic mean (UPGMA) based on hierarchical clustering at the OTU level. The picture on the right: sample distance heatmap analysis at the OTU level.

According to the UPGMA analyses, there were three geography-specific bacterial community clusters. The bacterial communities in AH1, AH2, and AH3 samples from Anhui were very similar to each other (Figure 4, group A). Similarly, the four samples from Yunnan (Figure 4, group C) and four samples from Hunan (Figure 4, group D) were respectively clustered together based on their geographic locations. However, for samples from the northeast (Heilongjiang and Jilin) and eastern China (Shandong), their samples were inter-mixed. For example, three samples each from HRB and DQ were clustered in group B, but the remaining sample from HRB and DQ (one each from these two regions) were clustered with those from other geographic areas (Figure 4). Heatmap analyses showed similar results. Groups A, B, C, D, and E described in the UPGMA graph were similarly found in the heat map. Within groups A, B, and C, the sample distances at the OTU level were close except DQ_3. In contrast, within group D (i.e., four samples within the HN region), the distances among the four biological replicates at the OTU level were far apart from each other (Figure 4).

### Functional prediction analysis of bacterial communities

**FIGURE 5:**
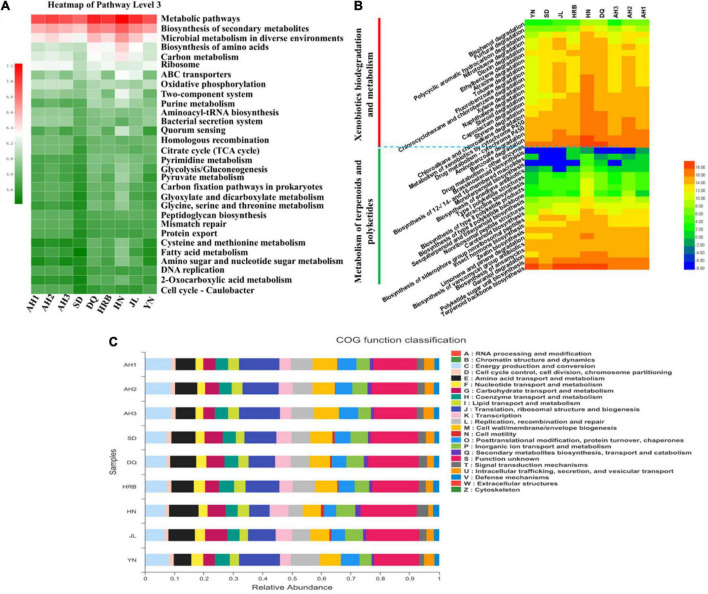
Comparison of predicted KEGG pathways and COG functions among nine geographic populations of the hemp flea beetle *P. attenuata* in China by PICRUSt2. **(A)** The relative abundance of KEGG pathways prediction based on level 3. **(B)** The relative abundance of metabolism pathways. The inferred metabolic pathways are shown with xenobiotics biodegradation and metabolism and metabolism of terpenoids and polyketides in the pathway level 2, which belong to the metabolism in pathway level 1. **(C)** The relative abundance of predicted COG functions.

To understand the potential roles of the bacterial community at the nine local populations of the hemp flea beetle *P. attenuata* in China, Kyoto Encyclopedia of Genes and Genomes (KEGG) pathways and the Cluster of Orthologous Groups (COG) functions were predicted (Figure 5). We found that the highest relative abundance of KEGG pathways at level 3 prediction was “metabolic pathways,” “biosynthesis of secondary metabolites,” and “microbial metabolism in diverse environments” (Figure 5A). The relative abundances of “xenobiotics biodegradation and metabolism,” “metabolism of terpenoids,” and “polyketides metabolic pathways” at level 2 pathway predictions, which belong to the “general metabolism” in level 1 pathway prediction were overall high (Figure 5B). The relative abundance of predicted COG functions was shown in Figure 5C. The functional category with the highest abundance was “function unknown.” The main metabolism functional features included translation, ribosomal structure and biogenesis, energy production and conversion, cell wall/membrane/envelope biogenesis, replication, recombination and repair.

### Comparison of fungal communities

The composition of the fungal communities within and among the nine geographic populations of the hemp flea beetle *P. attenuata* in China was investigated by high-throughput metabarcoding ITS1 sequencing. The finalized ITS sequencing data of the total sample contained 2,458,552 reads with an average length of 228 bp. In total, 910 OTUs were identified. These OTUs belonged to 9 phyla, 308 genera and 464 species (Table 2 and Supplementary Table 3). After rarefaction to the sample with the lowest number of sequence reads, 746 OTUs were clustered, attributed to 9 phyla and 277 genera and 410 known species for the nine *P. attenuata* populations (Table 2 and Supplementary Table 4). Rarefaction curves showed that all 36 samples approached asymptote, consistent with sufficient sequencing depths for each of the 36 samples analyzed here for fungal diversity (Supplementary Figure 4).

**FIGURE 6:**
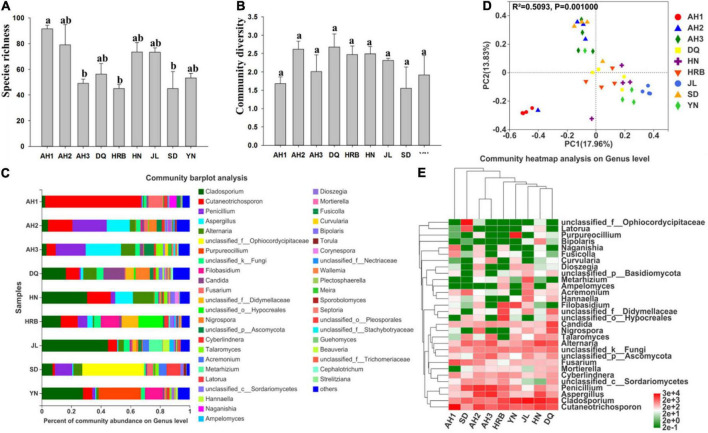
Fungal community composition within and among the nine geographic populations of the hemp flea beetle *P. attenuata* in China. **(A)** Histogram of species richness (number of OTUs). **(B)** Histogram of community diversity measured by Shannon index. Letters above each population indicate significant differences (one-way ANOVA, Tukey test, *P* < 0.05, see Table 3) in the mean values. **(C)** Relative abundance of fungal genera in different populations. **(D)** Principal components analysis (PCoA) plots of fungal communities based on the Bray-Curtis distance. Each symbol or color represents a sample. The significant differences in beta diversities were analyzed using Adonis analysis with 999 permutations, *P* < 0.05. **(E)** Heatmap showing the relative abundance of top thirty genera. Hierarchical cluster analysis was based on the Bray – Curtis distance with average method.

There were significant differences in fungal species richness (observed OTUs) between AH1 and AH3, HRB, SD (Figure 6A and Table 2). There was no significant difference in fungal community diversity (Shannon) based on Alpha-diversity analyses among the nine local populations of the hemp flea beetle *P. attenuata* in China (Figure 6B and Table 3). At the phylum level, Ascomycota and Basidiomycota were found to constitute the main members of the fungal community (Supplementary Figure 5). At the genus level, OTUs of AH1 were mainly annotated to *Cutaneotrichosporon* (64.86%) and *Fusarium* (9.80%); OTUs of AH2 were mainly annotated to *Cutaneotrichosporon* (16.54%), *Aspergillus* (15.57%), and *Nigrospora* (5.36%); OTUs of AH3 were mainly annotated to *Aspergillus* (23.88%) and *Alternaria* (9.65%); OTUs of SD were mainly annotated to *unclassified_f__Ophiocordycipitaceae* (41.79%) and *Latorua* (9.42%); OTUs of DQ were mainly annotated to *Cladosporium* (16.35%), *Candida* (14.31%), and *Cutaneotrichosporon* (9.18%); OTUs of HRB were mainly annotated to *Cladosporium* (12.86%), *Cutaneotrichosporon* (12.52%), *unclassified_f__Didymellaceae* (11.26%), *unclassified_o__Hypocreales* (16.52%), and *Filobasidium* (12.42%); OTUs of JL were mainly annotated to *Cladosporium* (44.79%) and *Metarhizium* (9.09%); and OTUs of YN were mainly annotated to *Cladosporium* (27.93%), *Purpureocillium* (28.65%), and *Filobasidium* (11.9%) (Figure 6C). The similarities and differences in composition and structure of the fungal communities of all samples were investigated by Principal components analysis (PCoA). PCoA separated most field populations (ADONIS test with 999 permutations, *P* < 0.05) but not the DQ, HN, HRB, and YN (Figure 6D). We selected the fungal genera with the top thirty abundance ratios and drew heat maps based on their relative abundance within different populations (Figure 6E). From the overall distribution of the fungal community at different populations, *Cladosporium*, *Cutaneotrichosporon* and *Aspergillus* were the dominant genera (Figure 6E and Supplementary Figure 6) across most of the samples but not the AH3 and SD populations. *Unclassified_f__Ophiocordycipitaceae* (41.79%) was abundant in SD, relatively rare in other populations. The abundance ratio of *Purpureocillium* in YN was significantly higher than those of the other populations (Figure 6E and Supplementary Figure 6).

### Abundance of fungi across samples

**FIGURE 7:**
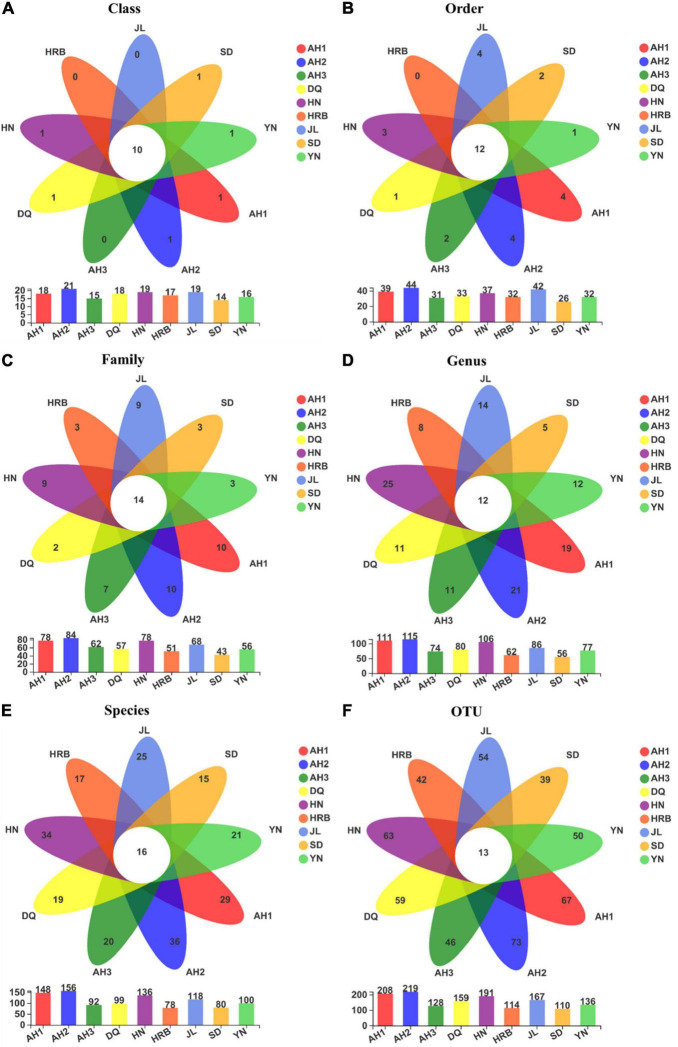
**(A–F)** Overlap in fungal community composition at different taxonomic levels among the nine geographic populations of the hemp flea beetle *P. attenuata* in China, with the number of fungi unique to each population in the petal and the number of fungi common to all populations in the center. Below is a bar chart of the total number of species in each population at selected taxonomic level.

Fungal community overlap at different taxonomic levels among the nine local populations of the hemp flea beetle *P. attenuata* in China is shown in Figure 6. At the OTU level, the AH2 population has the highest fungal species richness (Figure 7). Overall, there were 13 shared fungal OTUs among all geographic samples, and the unique number of OTUs of the AH1, AH2, AH3, DQ, HN, HRB, JL, SD, and YN were respectively 67, 73, 46, 59, 63, 42, 54, 39, and 50 (Figure 7). At the other taxonomic levels, there were 10 shared Classes, 12 shared Orders, 14 shared Families, 12 shared Genera, and 16 shared known species among the nine geographic samples (Figure 7).

### Fungal community dissimilarity across samples

**FIGURE 8:**
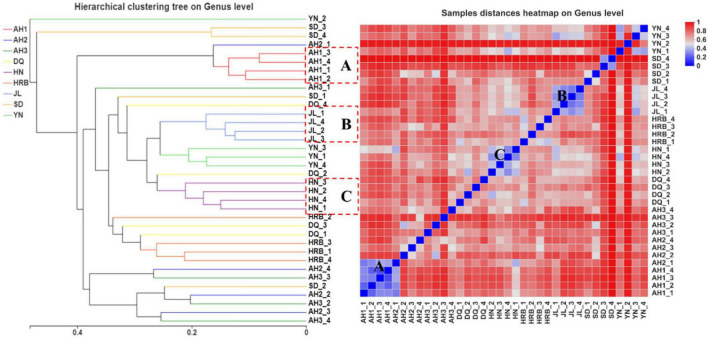
UPGMA (left) and heatmap (right) analyses of the fungal community in the nine geographic populations of the hemp flea beetle *P. attenuata* in China.

According to the UPGMA analyses, replicate samples within three of the nine geographic regions were clustered according to their geographic origins. Those included the AH1, JL, and HN samples respectively (Figure 8). For replicate samples from other six geographic locations, they were not uniformly clustered together based on their geographic locations. Instead, in most cases, 2−3 of the four samples from each location were clustered together while the remaining 1−2 showed more similar fungal assembly to samples from other geographic regions. Heatmap analyses showed a similar pattern as that shown on the UPGMA graph (Figure 8).

### Functional predictive analysis of fungal communities

**FIGURE 9:**
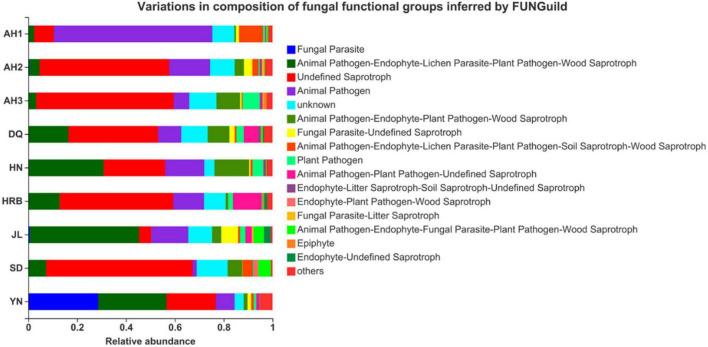
Comparison of predicted fungal functional groups among the nine geographic populations of the hemp flea beetle *P. attenuata* in China by FUNGuild.

To understand the potential roles of the fungal community among the nine local populations of the hemp flea beetle *P. attenuata* in China, putative fungal functions were predicted based on FUNGuild (Figure 9). The main fungal functional features included (i) animal pathogen-endophyte-lichen parasite-plant pathogen-wood saprotroph, (ii) undefined saprotroph, (iii) animal pathogen, and (iv) unknown.

## Discussion

In contrast to many other agricultural pests, little is known about *P. attenuata*, including its internal microbial community. Here, the compositions and structures of the microbiota communities of *P. attenuata* from fields across the hemp growth regions in China were surveyed by high-throughput sequencing of 16S rDNA gene for bacteria and ITS1 DNA fragment for fungi. We found that Proteobacteria (59.57−86.88%) and Bacteroidetes (2.52−35.21%) dominated the bacterial community across eight of the nine geographic samples (HN was the exception). The observed bacterial distribution pattern showed both similarities and differences with those in other insect species (Supplementary Figure 2). For example, in sap-feeding insects, such as aphids (Gauthier et al., 2015; Zhao et al., 2016), Proteobacteria was also the most abundant bacteria phyla. In many insect species of Lepidoptera, Diptera, Coleoptera, Hymenoptera, Hemipter and mites, the most prevalent phyla were Proteobacteria and Firmicutes (Zhang et al., 2020). Species in phylum Proteobacteria are known to perform many important functions such as nitrogen fixation, metabolisms of critical nutritional components (including sugars and proteins), insecticide resistance, and protection against parasites and pathogens in fruit flies, aphids, and moths (Zhang et al., 2020). In our study, we found that the highest relative abundances of KEGG pathways prediction at “level 3” were “metabolic pathways,” “biosynthesis of secondary metabolites,” and “microbial metabolism in diverse environments” (Figure 5). Therefore, Proteobacteria species likely play an important role in the nutrition and metabolism of *P. attenuata*.

At the genus level, dominant bacterial genera were *Rickettsia* (Proteobacteria), *Wolbachia* (Proteobacteria), *Candidatus_Brownia* (Bacteroidetes), *Enterobacter* (Proteobacteria), *Pantoea* (Proteobacteria), and *Rhodococcus* (Actinobacteria) (Figure 2C). This observed pattern is different from those reported in several other insects. For example, in *Dendroctonus* beetles, the dominant bacterial genera were *Erwinia*, *Serratia*, *Klebsiella*, *Rhodococcus*, and *Sphingomonas* (Xu et al., 2019).

For the first time, *Rickettsia* and *Wolbachia* infections were observed in *P. attenuata. Wolbachia* belongs to alpha-proteobacteria and strains in this genus are common intracellular parasites in arthropods and nematodes. It’s estimated that up to 70% of all insect species may contain *Wolbachia* (Kozek and Rao, 2007). These endosymbionts can infect many host tissues but the dominant tissues are those involved in sexual reproduction. Indeed, *Wolbachia* bacteria have been found to alter host insect biology in several ways, including nutrient provision, feminization, male-killing, parthenogenesis, and sperm-egg incompatibility or cytoplasmic incompatibility (Werren et al., 2008). Cytoplasmic incompatibility refers to the inability of con-specific sperm and egg to form viable offspring and it occurs when a *Wolbachia* infected male mates with a female that is either uninfected or infected by a different *Wolbachia* strain (Yen and Barr, 1971). Cytoplasmic incompatibility reduces the reproductive success of uninfected females and promotes the spread of *Wolbachia* strains infecting females. While the major mode of their transmission is vertical, they can also spread horizontally between host species (Werren et al., 2008). Indeed, due to their significant roles, *Wolbachia* bacteria have been explored as a potential agent in the control of mosquito-borne diseases in humans because of their unique ability to invade host mosquito populations rapidly (Pan et al., 2012). Similar suggestions have been made for controlling plant pathogens and pests. For example, *Wolbachia* endosymbionts in the Asian citrus pest psyllid *Diaphorina citri* can repress the holin promoter activity in the alpha-proteobacteria “*Candidatus Liberibacter asiaticus*,” a citrus disease agent, by killing the bacteria and preventing the disease from spreading (Jain et al., 2017).

The endosymbiotic *Wolbachia* is closely related to another intracellular bacterial genus *Rickettsia* (Perlman et al., 2006; Duron et al., 2008). While *Rickettsia* are most noted for causing human diseases, including Rocky Mountain spotted fever and epidemic typhus, they are also widely distributed in insects and play diverse roles (Perlman et al., 2006; Karkouri et al., 2022). For example, *Rickettsia* have shown to provide nutrition for host insects, improve their ability to resist high temperature and natural enemies, and significantly enhance the resistance of whitefly to infection by the bacterial pathogen *Pseudomonas syringae* (Zhang et al., 2021). Similar to those of *Wolbachia, Rickettsia* bacteria have shown to be capable of manipulating the reproductive behavior of host arthropods, including fertility, male-killing, and parthenogenesis (Perlman et al., 2006). In our study, the dominant bacterial genus in AH1, AH2, AH3, and SD were *Rickettsia*, the dominant bacterial genus in DQ, HRB, and YN were *Wolbachia*, and the dominant bacterial genera in JL was *Rickettsia* and *Wolbachia*. At present, the detailed roles of *Wolbachia* and *Rickettsia* to their host *P. attenuata* are not known. If they are found to play key roles in the survival and reproduction of *P. attenuata* in these regions, a potential novel control strategy against hemp flea beetles can be developed by targeting these endosymbiotic bacteria within these beetles.

Interestingly, the bacterial species richness and community diversity of the HN samples were significantly higher than those of other populations (Figures 2A,B). Furthermore, principal co-ordinate analysis showed that the community structures were similar among the four biological replicates within the HN region (Figure 2D). Therefore, the high bacterial species richness and community diversity of the HN samples were likely due to the uniqueness of the ecological niche at this site. Previous studies have shown that the microbial community in insects reflects the environmental microbial populations which in turn impact the insect host’s biology (Xu et al., 2019). The sampling site within Hunan province is rich in organic matter and has a climate different from other eight sites. Specifically, the site has had a long history of growing industrial hemp and at the site, the summers are hot, humid, and overcast while the winters are cold, humid, windy, and often cloudy. Over the course of the year, the temperature typically varies from 8°C to 33°C and is rarely below 4°C or above 36°C. At present, how these two features might have contributed to its distinct hemp beetle bacterial community in HN remain to be investigated. Additional sampling at sites with similar climates to those analyzed here as well as their corresponding soil samples are needed to determine the relative contributions of climate and soil niche to differences in the microbiome of hemp flea beetles.

The genus *Lactococcus* was abundant in the JL samples (20.64%) but rare in the other populations (<0.4%). *Lactococcus* is a genus of lactic acid-producing bacteria where only a single product lactic acid is produced during glucose fermentation. The broad distribution of *Lactococcus* bacteria in the Jilin samples suggested the potential importance of this fermentative pathway for this population of hemp flea beetles. In addition, this metabolic uniqueness of the Jilin samples may reflect other host phenotypic differences such as pesticide resistance. For instance, insecticide- resistant strains of the diamondback moth *Plutella xylostella* contained more abundant bacteria belonging to *Lactobacillales, Pseudomonadales*, and *Xanthomonadales* but fewer *Enterobacteriales* than an insecticide-susceptible strain (Xia et al., 2013). The genus *Pantoea* was abundant in the SD population (33.21%) and was rare in other populations (<2%). *Pantoea* is a highly diverse genus that can cause plant diseases and human diseases but also have functions in habitat restoration and pesticide degradation (Walterson and Stavrinides, 2015). It’s possible that *Pantoea* in the SD populations functioned similarly to those found in plants and humans. At present, how bacteria in genera *Lactococcus* and *Pantoea* might contribute to trait differences among *P. attenuata* populations and among the host plant *Cannabis sativa* are unknown.

Aside from comparing bacterial diversity, we also investigated the mycobiota of the *P. attenuata* samples. The dominant fungal phyla in our samples belonged to Ascomycota and Basidiomycota (Supplementary Figure 5). This result was not surprising because the great majority of known terrestrial fungi are in Ascomycota and Basidiomycota (Stajich et al., 2009), including molds, mushrooms, rusts, smuts, and yeasts. Species in Ascomycota and Basidiomycota play important ecological and economical roles, including making essential contributions to the bio-product industry, nutrient cycling, antibiotic production, and as animal, human and plant pathogens (Pei et al., 2019). In our study, the fungal communities of all samples of hemp flea beetles were dominated by common environmental fungi in Ascomycota and Basidiomycota. This result is consistent with the hypothesis that environmental fungal community is a key determinant of the fungal community within hemp flea beetles.

Our analyses revealed that the total number of known bacterial and fungal species was greater than the total number of bacterial and fungal OTUs. This result is not surprising because the OTUs were defined based on a 97% sequence similarity cutoff for both the bacterial and fungal datasets while many currently known closely related sister species had higher sequence similarities than 97% at these two barcode loci (Edgar, 2013; Xu, 2016, 2020). Given that there are unclassified novel taxa in both the bacterial and fungal datasets, the true numbers of bacterial and fungal species in our samples of *P. attenuata* are likely much higher than what we found here. High coverage metagenome sequencing coupled with culture-based surveys will help generate more robust estimates of bacterial and fungal diversities in these samples (Xu, 2020). The obtained microbial cultures will also allow direct tests of their potential roles in the survival and reproduction of hemp flea beetles.

We note that even though several geography-specific microbial community clusters were found in both the bacterial and fungal datasets, such clusters could be due to differences in other factors associated with the specific geographic regions, including climate, host cultivars, soil conditions, and host pest genotypes. Indeed, the four broad geographic regions (i.e., Northeast China, East-central China, Central-south China, and Southwestern China) analyzed here are known to differ from each other in at least one of the above factors. In addition, we observed samples in several geographic regions that were not clustered according to their geographic origins. At present, the reasons for such differences are not known. However, fine-scale heterogeneity in soil microflora, hemp clones/cultivars, health status (e.g., microbial infections) of sampled host hemp plants, and the host plant microbiome within crop fields could all have contributed to microbiome differences among hemp flea beetles sampled from different parts of a field. Fine-scale sampling of diverse microbiomes from the soil, host plants, and hemp flea beetles are needed in order to test their specific effects on the microbiome structures of hemp flea beetles.

## Conclusion

The present study revealed the bacterial and fungal communities associated with hemp flea beetles across hemp production regions in China. Our analyses demonstrated that there were differences in microflora among field populations in different locations, which likely reflect their adaption to their local environments and to potential phenotypic differences among their host beetles. More research is required to explore the detailed roles of these microbes in *P. attenuata*, including how environmental factors may impact their interactions. One such approach is to isolate specific microbe and then test their effects on hemp flea beetles by inoculating them to hemp flea beetles from diverse locations. Similarly, the identification of widespread and dominant bacteria in genera *Wolbachia* and *Rickettsia* and several other bacteria suggests potentially key roles of these bacteria for hemp flea beetles, especially on the reproduction of these pest insects (Duron et al., 2008). Regarding the potential role of *Wolbachia* on cytoplasmic incompatibility in hemp flea beetles, additional research is needed on the distribution and genetic diversity of *Wolbachia* strains among hemp flea beetles from within and between geographic regions. Furthermore, genetic crosses between hemp flea beetles uninfected or infected with the same or different *Wolbachia* strains need to be analyzed. Such data will be crucial for evaluating whether these bacteria could be potentially used as biocontrol agents against these pest beetles in hemp fields and greenhouses.

## Data availability statement

The datasets presented in this study can be found in online repositories. The names of the repository/repositories and accession number(s) can be found in the article/Supplementary material.

## Author contributions

LG and JX conceived and designed the experiments and wrote the manuscript. LG and CT performed the experiments. LG analyzed the data. All authors contributed to the reagents, materials, and analysis tools.

## Footnotes

1
www.majorbio.com

## Supplementary material

Supplementary Table 1 The original bacterial OTUs based on 16S rRNA sequences across nine geographic populations of the hemp flea beetle *P. attenuata* in China.

Supplementary Table 2 The bacterial OTUs based on 16S rRNA sequences after rarefaction to the lowest number of reads among the 36 samples across the nine geographic populations of the hemp flea beetle *P. attenuata* in China.

Supplementary Table 3 The original fungal OTUs based on ITS sequences across nine geographic populations of the hemp flea beetle *P. attenuata* in China.

Supplementary Table 4 The fungal OTUs based on ITS sequences after rarefaction to the lowest number of reads among the 36 samples across the nine geographic populations of the hemp flea beetle *P. attenuata* in China.

Supplementary Table 5 The raw data of the 16S rRNA read counts of bacteria across nine geographic populations of the hemp flea beetle *P. attenuata* in China.

Supplementary Table 6 The raw data of diversity indices of bacteria based on 16S rRNA sequences across nine geographic populations of the hemp flea beetle *P. attenuata* in China.

Supplementary Table 7 The raw data of the ITS read counts of fungi across nine geographic populations of the hemp flea beetle *P. attenuata* in China.

Supplementary Table 8 The raw data of the diversity indices of fungi based on ITS sequences across nine geographic populations of the hemp flea beetle *P. attenuata* in China.
