# Supplementary material for: Bacterial and fungal communities within and among geographic samples of the hemp pest Psylliodes attenuata from China
Source: Front Microbiol. 2022 Sep 6;13:964735. doi: 10.3389/fmicb.2022.964735 (PMC9485832; doi:10.3389/fmicb.2022.964735)

**SUPPLEMENTARY MATERIAL**

**Figure S1 |** Rarefaction curves of OTUs clustered at 97% sequence identity with the 16S rRNA sequences from the hemp flea beetle *P. attenuata*. **A** Rarefaction curves of the original OTUs of the 16S rRNA. **B** Rarefaction curves of the OTUs of the 16S rRNA after rarefying according to the minimum sample sequence number. Sobs represents the observed number of species.


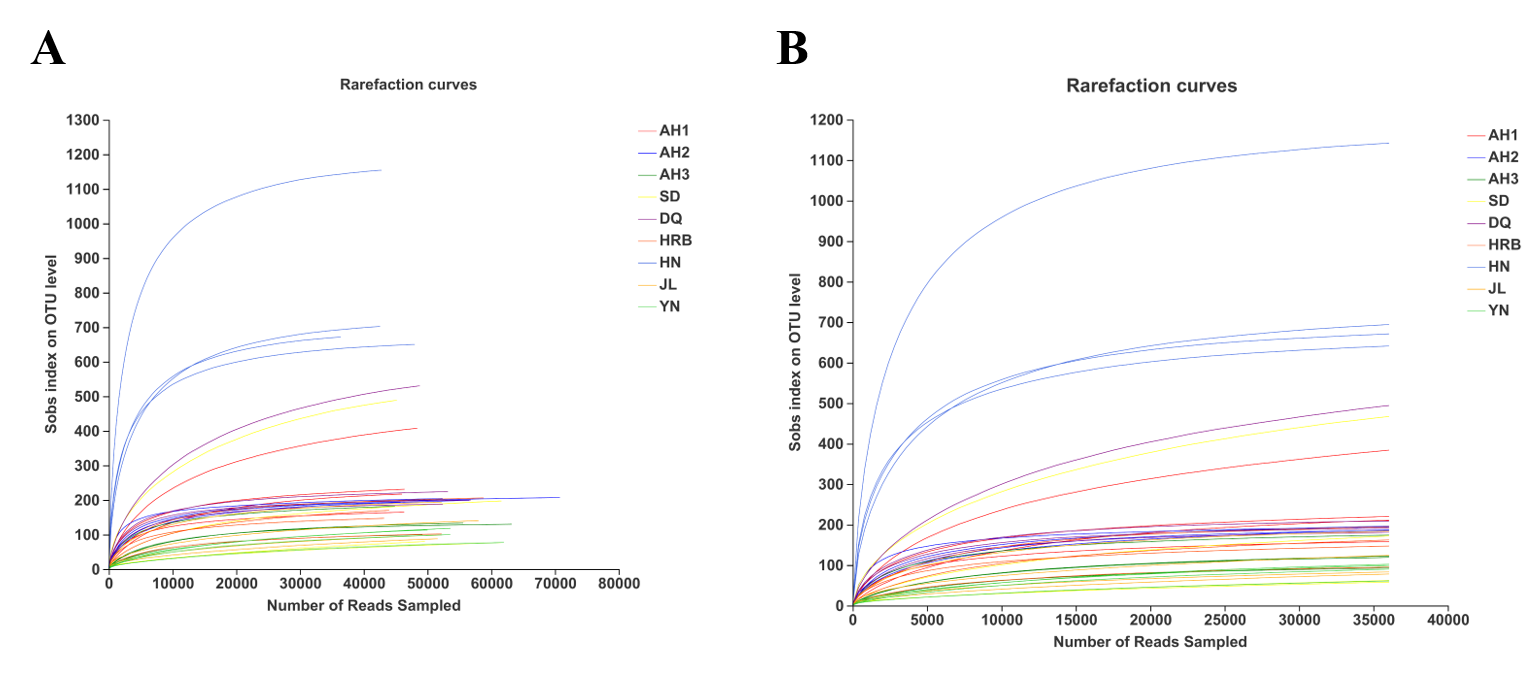


**Figure S2 |** Relative abundance of bacteria at the phylum level among nine geographic populations.


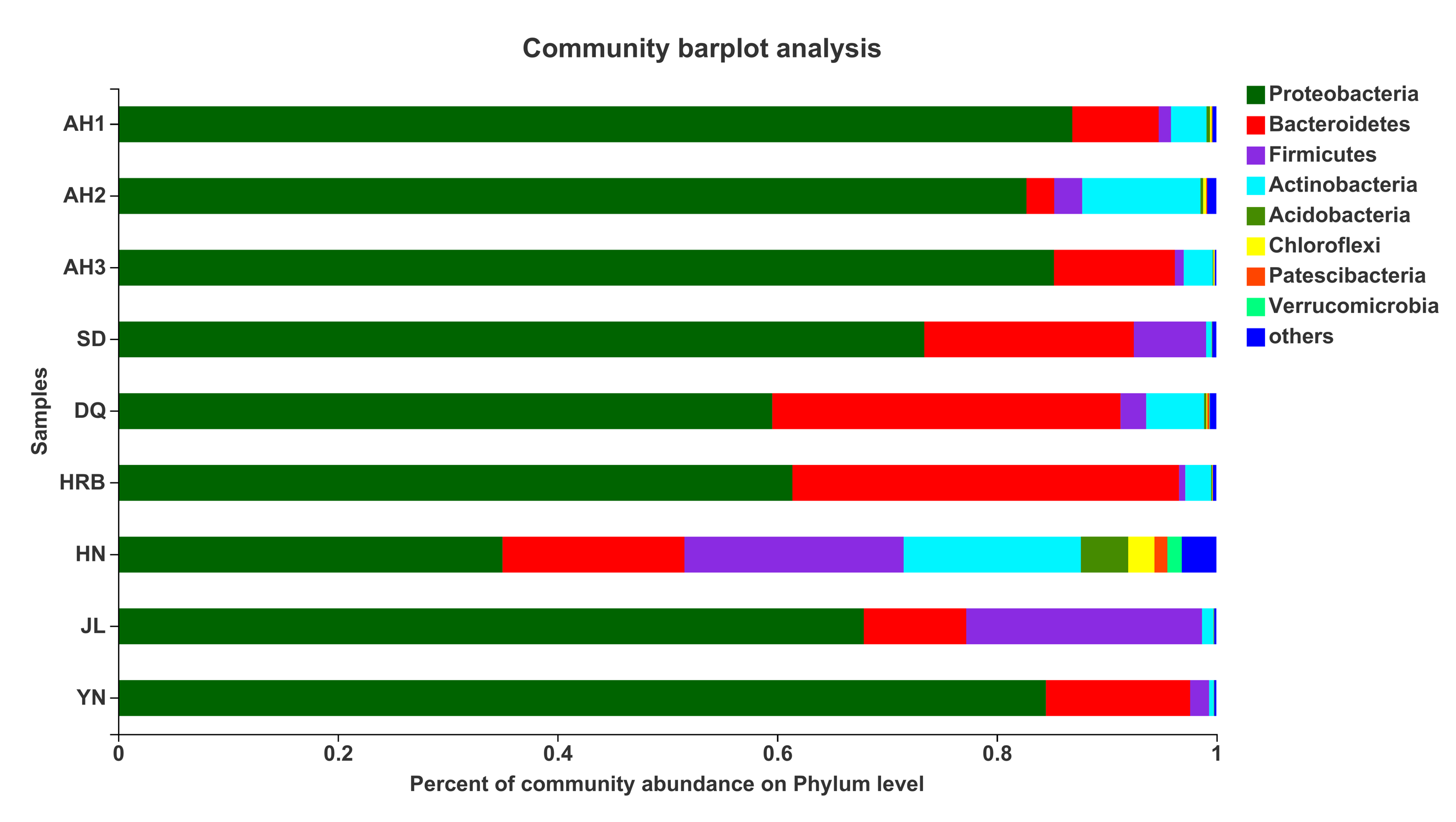


**Figure S3 |** Phylogenetic analysis of the top 50 most abundant bacterial OTUs in our sample at the genus level and their distributions among the nine geographic populations. The phylogenetic evolutionary tree is on the left, in which each branch represents a species and the branches are colored according to the higher taxonomic level to which the species belongs. The length of the branches is the evolutionary distance between the two OTUs, that is, the degree of species difference. The bar chart on the right shows the proportion of Reads among the nine geographic populations.


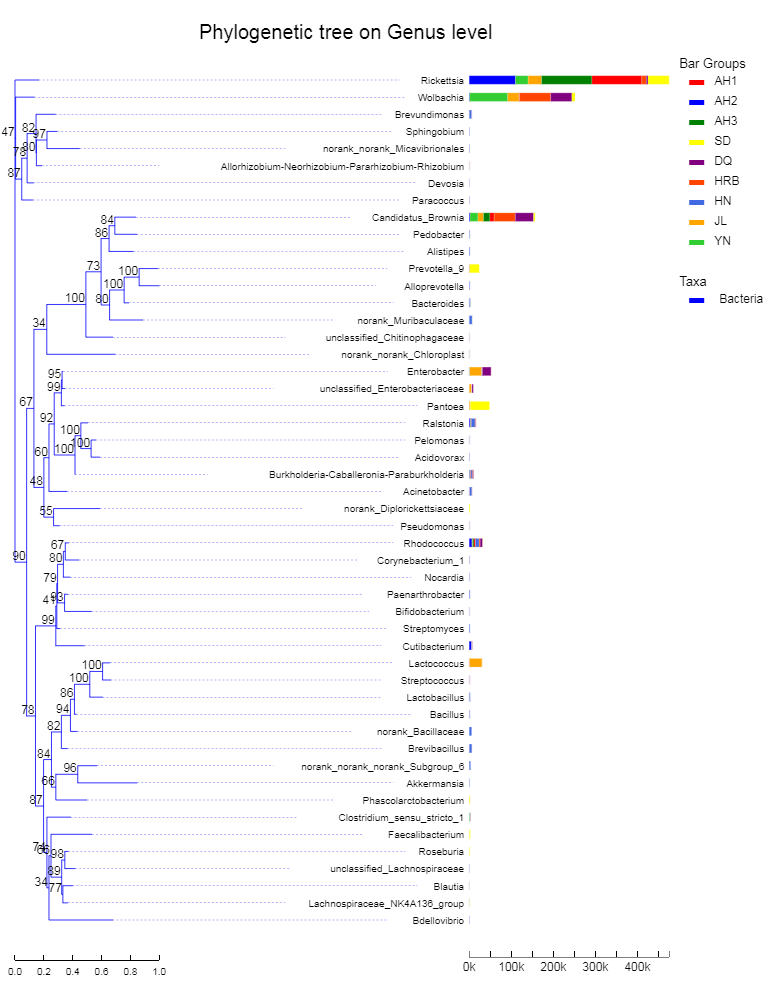


**Figure S4 |** Rarefaction curves of OTUs clustered at 97% sequence identity with the ITS sequences from the hemp flea beetle *P. attenuata*. **A** Rarefaction curves of the original OTUs of the ITS. **B** Rarefaction curves of the OTUs of the ITS after rarefying according to the minimum sample sequence number. Sobs represents the observed number of species.


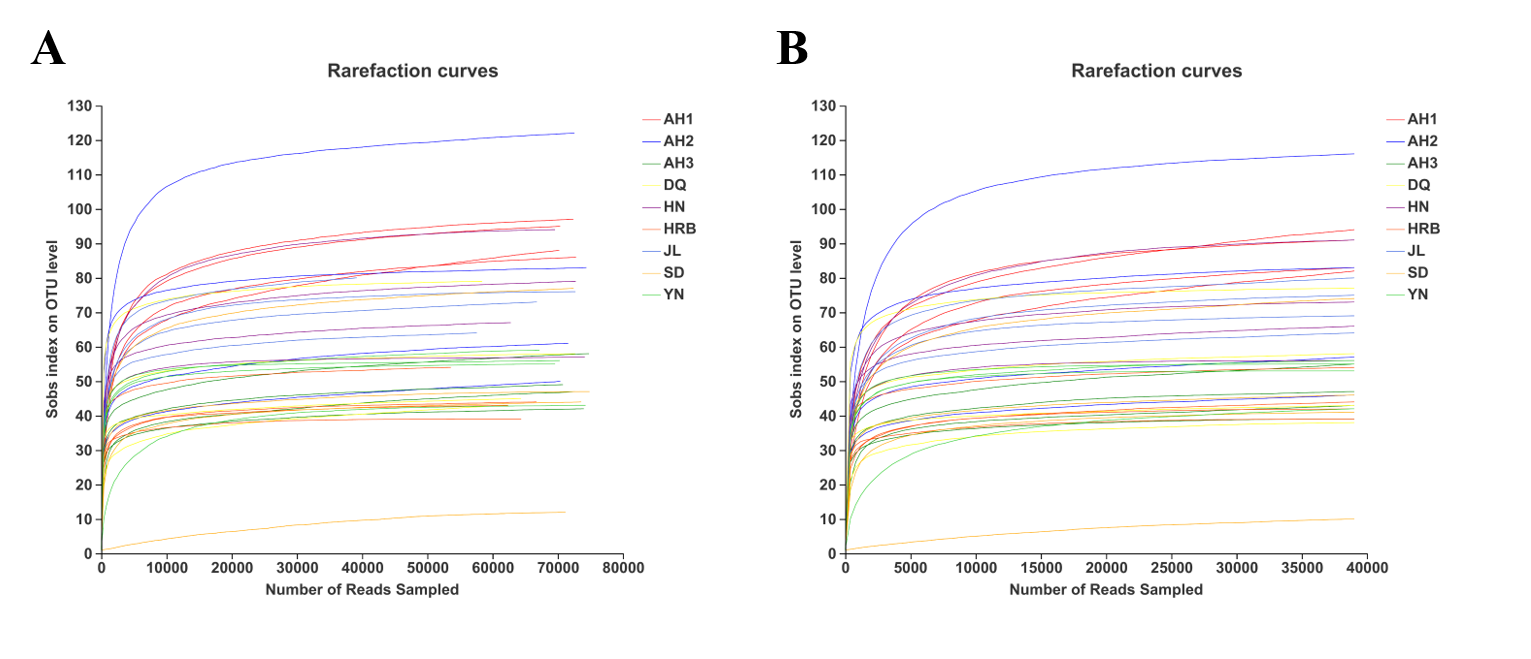


**Figure S5 |** Relative abundance of fungal OTUs at the phylum level among the nine geographic populations.


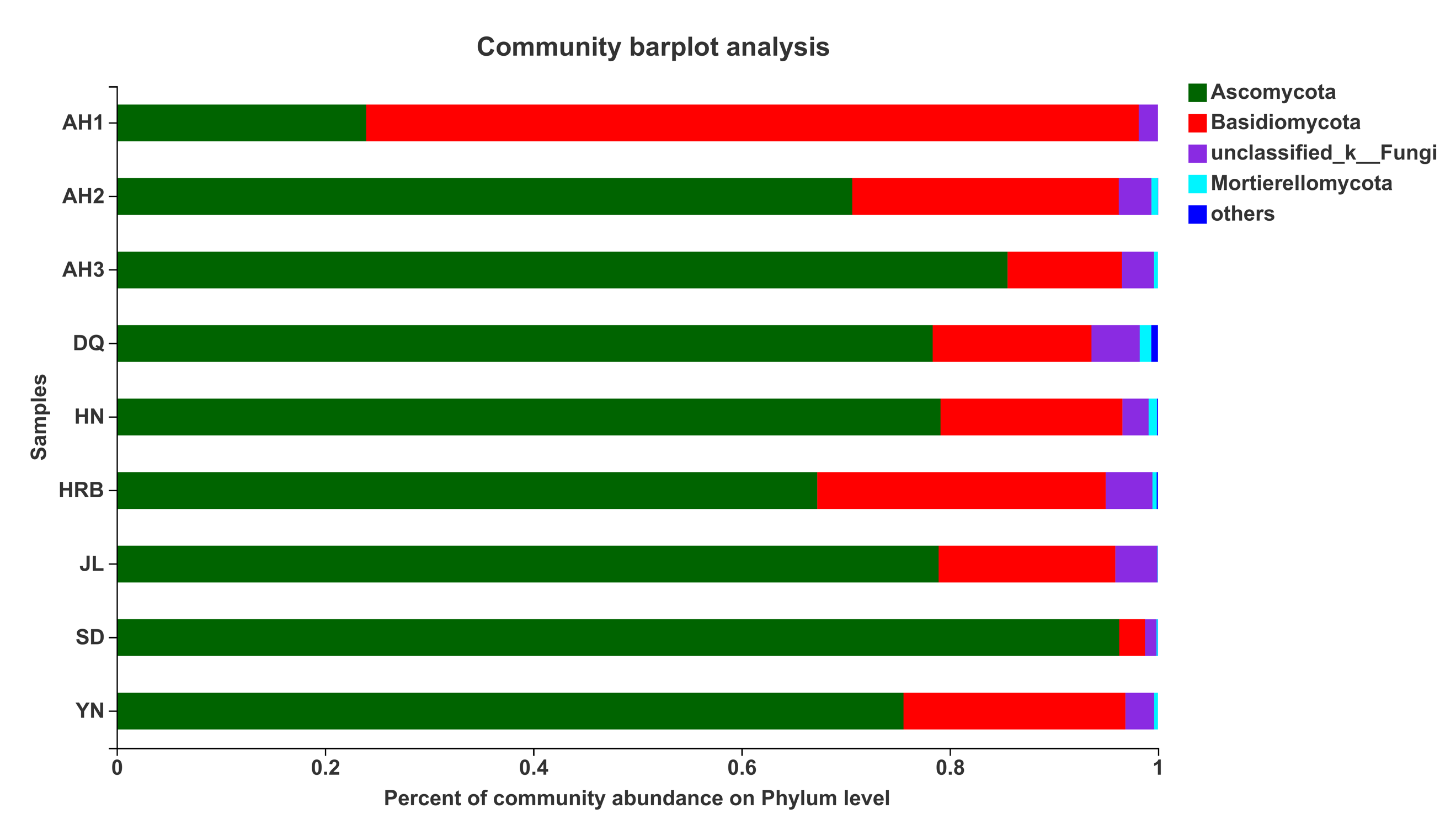


**Figure S6 |** Phylogenetic analysis the top 50 most abundant fungal OTUs in our sample at the genus level and their distributions among the nine geographic populations. The phylogenetic evolutionary tree is on the left, in which each branch represents an OTU and the branches are colored according to the genus level to which the OTU belongs. The length of the branches is the evolutionary distance between the two OTUs. The bar chart on the right shows the proportion of Reads among the nine geographic samples.


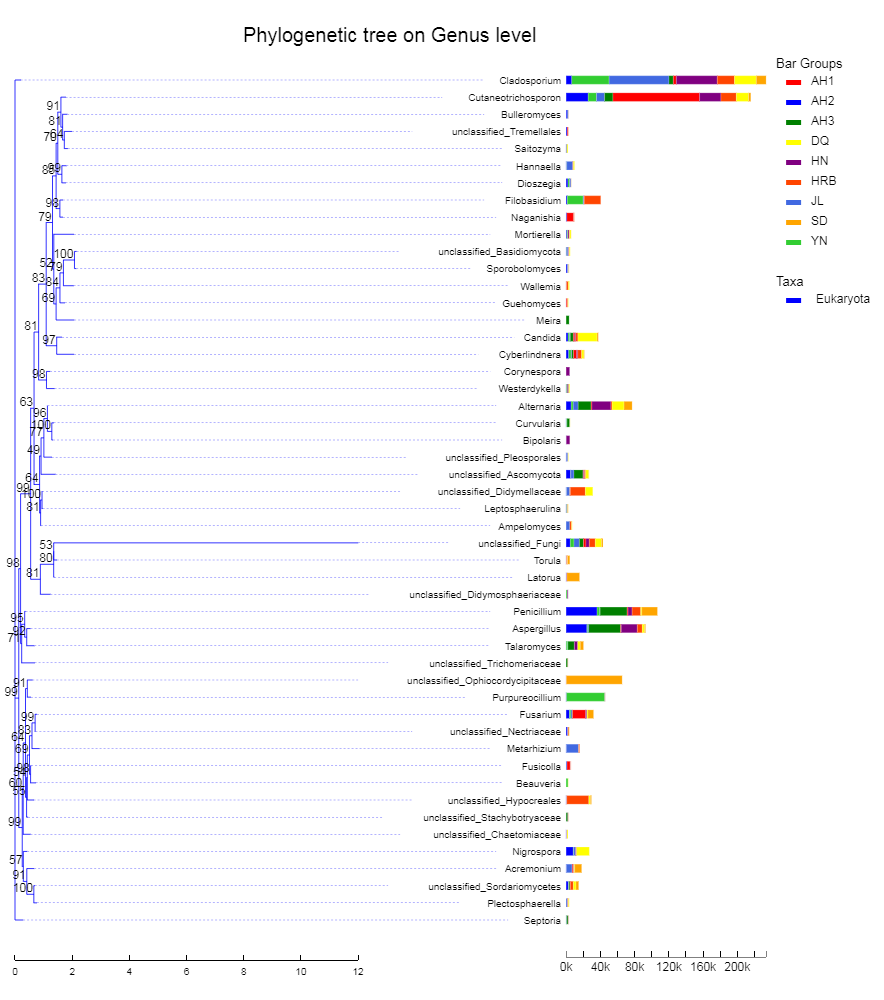

Supplement: Supplementary file 2 [file Data_Sheet_1.docx]
